# Association between ambient air pollution exposure and infants small for gestational age in Huangshi, China: a cross-sectional study

**DOI:** 10.1007/s11356-019-06268-7

**Published:** 2019-09-06

**Authors:** Jiayuan Hao, Faxue Zhang, Dieyi Chen, Yanyun Liu, Lina Liao, Cui Shen, Tianyu Liu, Jingling Liao, Lu Ma

**Affiliations:** 1 Department of Healthcare Management, School of Health Sciences, Wuhan University, Wuhan, 430071 China; 2 Huangshi Maternity and Children’s Health Hospital of Edong Healthcare Group, Huangshi, Hubei Province China; 3 Department of Public Health, Wuhan University of Science and Technology School of Medicine, Wuhan, China; 4 Global Health Institute, Wuhan University, Wuhan, 430071 China

**Keywords:** Air pollution, Particulate matter, Nitrogen dioxide, Sulfur dioxide, Small for gestational age, Adverse pregnancy outcome

## Abstract

Small for gestational age (SGA) is defined as intrauterine growth retardation or small sample, referring to the 10th percentile of birth weight lower or two standard deviations less than the average weight at the same gestational age. SGA infants bring great economic and psychological burdens to families and society. The association between exposure to air pollution and SGA in underdeveloped cities with poor air quality remains unclear. Thus, this study is conducted to estimate the effects of maternal exposure to air pollutants on SGA numbers. Birth information was collected from the Huangshi Maternity and Children’s Health Hospital from January 1st to December 31st in 2017. Data of pregnancy exposure were accessed using stationary monitors. These data included particulate matter less than or equal to 10 μm in aerodynamic diameter (PM_10_), particulate matter less than or equal to 2.5 μm in aerodynamic diameter (PM_2.5_), nitrogen dioxide (NO_2_), and sulfur dioxide (SO_2_). Multivariate logistic regression models were performed to estimate the association between ambient air pollution and the risk of SGA during different exposure windows. It was found that a 1 μg/m^3^ increase in air pollution concentrations during the entire pregnancy was associated with a higher risk of SGA, with an adjusted odds ratio (OR) and 95% confidence interval (CI) of 1.055 (1.035–1.076), 1.084 (1.053–1.116), 1.000 (0.953–1.049), and 1.051 (0.968–1.141) for PM_10_, PM_2.5_, NO_2_, and SO_2_, respectively. Thus, it is suggested that exposure to air pollution is associated with an increased risk of SGA. The effects of PM_10_ and PM_2.5_ were more stable than NO_2_ and SO_2_.

**Electronic supplementary material:**

The online version of this article (10.1007/s11356-019-06268-7) contains supplementary material, which is available to authorized users.

## Introduction

Small for gestational age (SGA) is defined as intrauterine growth retardation or small sample, and it refers to the 10th percentile of birth weight lower or two standard deviations less than the average weight at the same gestational age (Ding et al. 2013; Khambalia et al. 2017; Lefebvre and Samoilenko 2017). Similarly, appropriate size for gestational age (AGA) is known as a birth weight within the 10th to 90th percentile of the reference value. SGA is associated with a higher risk of infant morbidity and mortality (Basso et al. 2006). In addition, it can lead to complications in later childhood, such as endocrine and metabolic disturbances (Clayton et al. 2007). However, the risk factors for SGA have not been fully identified, although there are some possible causes, such as teenage motherhood, previous preterm birth, and inadequate prenatal visits (Kildea et al. 2017).

Previous studies have illustrated that exposure to high concentrations of air pollution during pregnancy may decrease uterine blood flow and ultimately slow fetal growth (Browne et al. 2015; Kannan et al. 2006). Maternal exposure to air pollution during pregnancy may be one of the complex set of causes, which increases the risk of impaired fetal development and adverse birth outcomes, such as preterm birth and low birth weight (Lamichhane et al. 2015; Li et al. 2017). Whether there is potential association between air pollution and gestational age as a typical factor for fetal growth needs to be addressed.

China has been troubled with air pollution for many years, and the evidence remains inconsistent whether high levels of air pollutants affect the incidence of SGA. Although there are some analyses that have explored the association between air pollution and SGA, most of them have been conducted in developed countries or regions with relatively low air pollution (Michikawa et al. 2017; Rich et al. 2009b; Schlesinger et al. 2006; Wang et al. 2019). To our knowledge, there are no existing studies that have investigated the relationship between air pollution and SGA in central China. Huangshi is an industrial city in central China, and its economic growth has been attributed to mining and metallurgy in recent years (Zhan et al. 2017). Although the concentrations of air pollutants in Huangshi are lower than those in other industrial cities, such as Wuhan (Qian et al. 2016), air pollution exposure in Huangshi is still relatively higher than the air quality guidelines (AQG) issued by the World Health Organization (WHO 2005). For example, compared with the AQG guidelines, the 24-h mean concentrations of particulate matter less than or equal to 10 μm in aerodynamic diameter (PM_10_) and particulate matter less than or equal to 2.5 μm in aerodynamic diameter (PM_2.5_) exceeded the WHO standard for 327 days and 295 days, respectively, in 2017. There are many prefecture-level industrial cities in central China with low residential mobility, like Huangshi. However, due to subaverage economic and demographic factors, prefecture cities have often been ignored in previous studies. As pregnant women do not have access to advanced medical services during pregnancy as in developed areas, it has been suggested that there might be more severe birth outcomes when pregnant mothers are exposed to eternal environmental pollution.

We chose Huangshi as the study area to investigate whether there are associations between SGA and exposure to air pollution, including PM_10_, PM_2.5_, nitrogen dioxide (NO_2_), and sulfur dioxide (SO_2_). Logistic regression models were constructed in different quartiles to estimate the potential threshold effects. Previous studies have reported that different fetal sexes had different responses towards the environmental simulation in vivo and in vitro, suggesting that infant sex is related to the regulation of birth health (Al-Qaraghouli and Fang 2017; Catalano et al. 2014; Challis et al. 2013; Liu et al. 2018). We also conducted analysis among male infants and female infants during different exposure windows to compare the variation between the different sexes.

## Methods

### Study area

Huangshi (114°31′-115°30′ East, 29°30′-30°15′ North) is a prefecture-level city in the Hubei province of central China. The city covers an area of 4583 km^2^ with a population of 2,689,300 people in 2017. With a subtropical monsoon climate, the city’s weather is mild and humid. However, as an industrial city, Huangshi has high level of air pollutants according to the WHO guideline values (WHO 2005).

### Study population

**Fig. 1:**
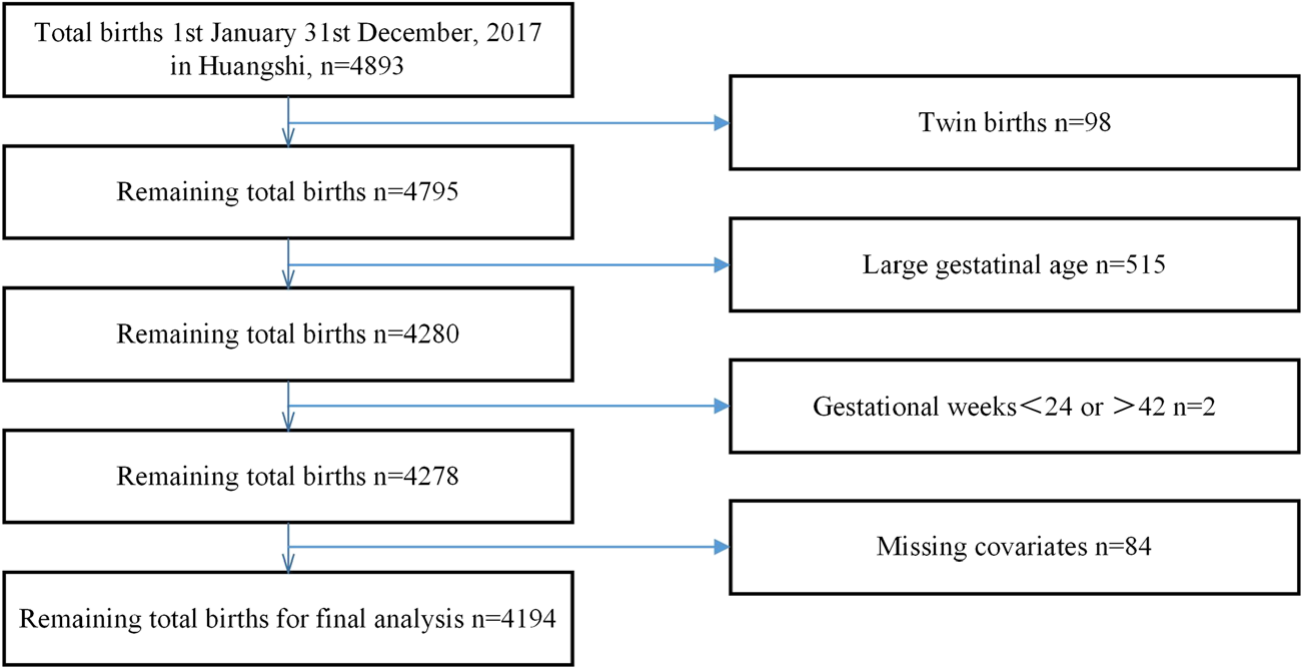
Flow chart of the study population selection

Information of mothers and their live births were collected from the Huangshi Maternity and Children’s Health Hospital from January 1, 2017, to December 31, 2017. The annual number of births in this hospital accounted for more than 33% of the city’s total delivery. The inclusion criteria included live singleton births after 24 completed weeks and within 42 weeks with complete covariates. In addition, infants whose birth weights were more than the 90th percentile of newborns needed to be excluded. Finally, 4194 singletons were identified (Fig. 1).

The covariates were collected from medical records documented by doctors and nurses after deliveries. The original data was recorded in medical report books and then transformed into electronic form. The collected variables included maternal age, parity, gestational age at birth, date of delivery, delivery mode, infant birth weight, infant sex, and maternal medical conditions during pregnancy including gestational hypertension and gestational diabetes mellitus. The date of conception was calculated using gestational weeks and the date of birth. The gestational age of infants in this study was defined using the adjusted last menstrual period and the delivery date. SGA was determined using a comparison of the weight ranges from the birth weight curve for China (Zhu et al. 2015).

### Exposure assessment

Air pollutant concentrations were obtained from the mirror website of the Environmental Protection Agency (https://www.aqistudy.cn/historydata/). The 24-h daily concentrations of PM_10_, PM_2.5_, NO_2_, and SO_2_ were collected. The time-varying average concentration approach was utilized to estimate individual exposure.

According to the different exposure windows, a pregnancy period was divided into the first trimester (1–12 weeks), the second trimester (13–27 weeks), and the third trimester (28 weeks to delivery) (Wu et al. 2009).

### Statistical analysis

The correlations between each air pollutant were calculated using the Pearson correlation. The chi-squared test for categorical variables and the independent sample *t* test for continuous variables were conducted for the univariate factor analysis of maternal and infant characteristics. A single-pollutant model and the calculated risk of SGA were conducted separately for each pollutant and pregnancy period.

To further explore the associations between air pollution and SGA, second-stage analyses were performed. Multiple logistic regression models adjusted for covariates were conducted to estimate the specified trimester association between air pollutants and SGA during the entire pregnancy, the first trimester, the second trimester, and the third trimester. All concentrations of pollutants were regarded as continuous variables, and the associations were shown as ORs and 95% CIs per 1 μg/m^3^ increase in PM_10_, PM_2.5_, NO_2_, and SO_2_.

Stratified analysis of the associations in subgroups for female and male infants was estimated during the different periods. To further explore the effect of air pollutants for their per interquartile range (IQR) increase and to observe the possible threshold effect, the association within subgroups of different quartiles compared with the reference group of the first quartile was assessed. Due to periodic variations in air pollutants, the effects of these seasonal variations on the association of air pollution and SGA were estimated.

Additionally, multi-pollutant models were conducted to explore the potential confounding effects of other pollutants on SGA during each pregnancy period. Due to the high correlation of PM_10_ and PM_2.5_, they were not included in one regression.

Statistical tests were two-sided, and a *P* value < 0.05 was considered statistically significant. All analyses were conducted using R 3.4.4 software with “tidyverse,” “rlist,” and “ggplot2” packages.

## Results

The demographic characteristics of the mother–infant pairs are shown in Table 1. A total of 4194 deliveries that met the criteria were collected from the Huangshi Maternity and Children’s Health Hospital in 2017. A total of 315 (7.5%) of these deliveries were SGA infants, and 3879 (92.5%) were AGA infants. The mean gestational age was 38.69 for SGA infants and 38.42 for AGA infants. A total of 84.2% of the mothers were less than 35 years old at the time of delivery, 2.1% were diagnosed with gestational hypertension, 3.7% were accompanied with gestational diabetes mellitus, and for 46.2% of them, this was their first delivery. Gestational age, maternal age, parity, and gestational diabetes mellitus were not statistically significantly associated with the risk of SGA. The SGA percentile was higher in infants whose mothers were diagnosed with gestational hypertension. In addition, a significantly higher proportion of SGA infants among female newborns were observed (*P* < 0.001).

**Table 1:** Summary characteristics of participants

Characteristics	SGA, *n* = 315	AGA, *n* = 3879	*t*/chi-square (*P*)
Gestational age (weeks, mean (SD))	38.69 ± 2.01	38.42 ± 1.91	0.403
Maternal age, *n* (%)			
≤ 24	74 (23.5)	812 (20.9)	0.598
25–29	104 (33.0)	1404 (36.2)	
30–34	84 (26.7)	1054 (37.2)	
≥ 35	53 (16.8)	609 (15.7)	
Parity, *n* (%)			
0	158 (50.2)	1780 (45.9)	0.144
≥ 1	157 (49.8)	2099 (54.1)	
Gestational hypertension, *n* (%)			
Yes	15 (4.8)	74 (1.9)	0.001
No	300 (95.2)	3805 (98.1)	
Gestational diabetes mellitus, *n* (%)			
Yes	9 (2.9)	147 (3.8)	0.400
No	306 (97.1)	3732 (96.2)	
Delivery mode, *n* (%)			
Vaginal	157 (49.8)	1874 (48.3)	0.601
Cesarean	158 (50.2)	2005 (51.7)	
Infant sex, *n* (%)			
Male	114 (36.2)	2243 (57.8)	< 0.001
Female	201 (63.8)	1636 (42.2)	

Chi-square test for categorical variables and independent sample *t* test for continuous variables

**Fig. 2:**
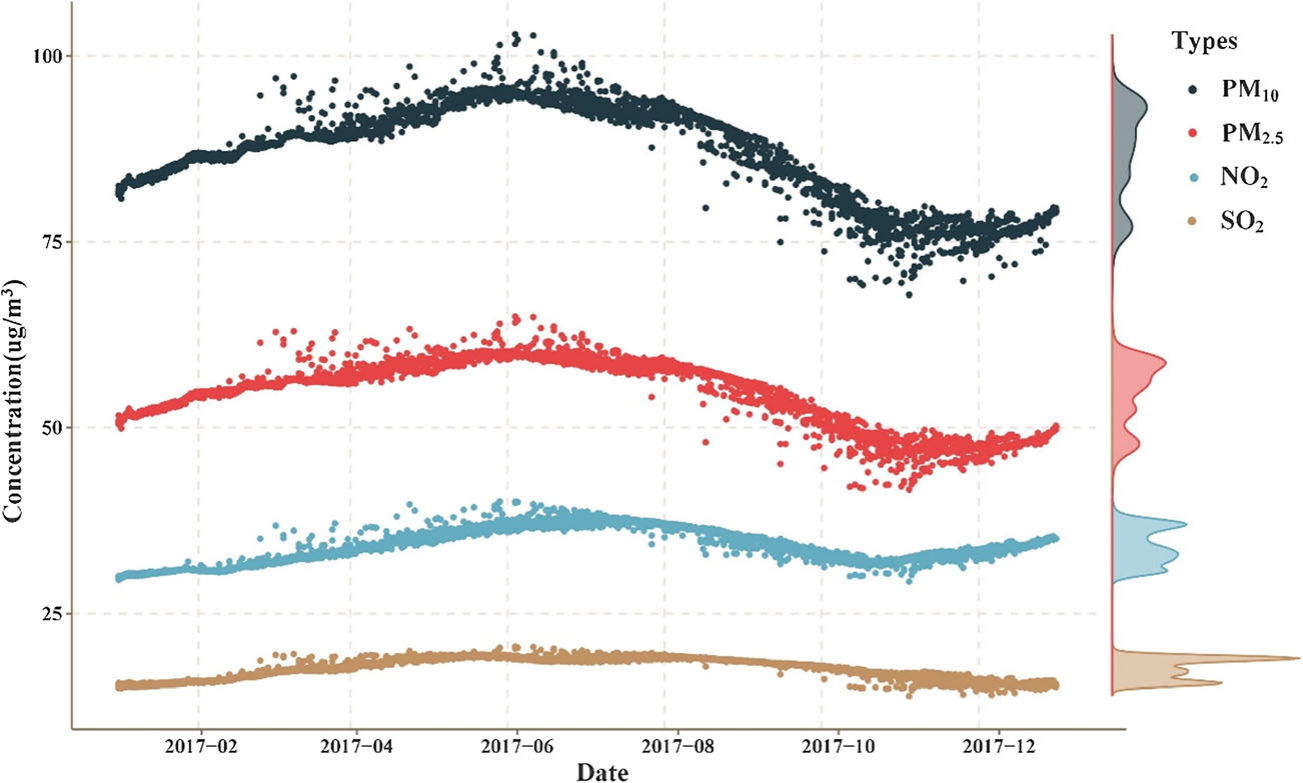
Mean concentrations of maternal exposure to four air pollutants including PM_10_, PM_2.5_, NO_2_, and SO_2_ during entire pregnancy

The average maternal exposures to the four air pollutants during the entire pregnancy are shown in Fig. 2. Women who delivered in summer had a higher exposure level to all the pollutants. The mean concentrations of PM_10_, PM_2.5_, NO_2_, and SO_2_ were 86.8 μg/m^3^, 55.3 μg/m^3^, 36.5 μg/m^3^, and 17.6 μg/m^3^, respectively. Compared with the AQG released by WHO (annual mean is 20 μg/m^3^ for PM_10_, 10 μg/m^3^ for PM_2.5_, and 40 μg/m^3^ for NO_2_; 24-h mean is 20 μg/m^3^ for SO_2_), the maternal exposure concentrations of PM_10_ and PM_2.5_ were far above those criteria. The correlations of each of two pollutants were calculated and are shown in Table S1. PM_10_ and PM_2.5_ were significantly associated with SO_2_, and Pearson’s correlation coefficients were 0.804 and 0.788, respectively. NO_2_ was less strongly correlated with the solid pollutants, and Pearson’s correlation coefficients for PM_10_ and PM_2.5_ were 0.660 and 0.644, respectively.

The crude ORs and adjusted ORs (adjusted for maternal age, parity, gestational hypertension, gestational diabetes mellitus, delivery mode, and infant sex) with 95% CI for each air pollutant and pregnancy period are presented in Table 2. The variation between crude ORs and adjusted ORs was subtle. Exposure to PM_10_ and PM_2.5_ was significantly associated with a risk of SGA during the entire pregnancy (aOR [95% CI], 1.055 [1.035–1.076] for PM_10_ and 1.084 [1.053–1.116] for PM_2.5_), and the adverse effects of PM_2.5_ were stronger than those of PM_10_. The effects of NO_2_ and SO_2_ were statistically significant during the first and third trimesters. For all of the pollutants, the highest ORs occurred during the third trimester (aOR [95% CI], 1.018 [1.012–1.024], 1.022 [1.013–1.030], 1.037 [1.019–1.056], and 1.117 [1.082–1.153] for PM_10_, PM_2.5_, NO_2_, and SO_2_, respectively). The lowest ORs appeared during the first trimester, showing as an inverse effect on SGA (aOR [95% CI], 0.979 [0.971–0.987], 0.976 [0.966–0.986], 0.959 [0.945–0.973], 0.887 [0.857–0.919], respectively). The effects of air pollutants per IQR increase are presented in Table 3, with the first quartile as a reference. In the case of PM_10_ and PM_2.5_, the risk of SGA through the entire pregnancy increased; however, there were no observable remarkable variations among the different quartiles. The effects of PM_10_ and PM_2.5_ were obviously strengthened for per IQR rise in pollutants during all the different trimesters. NO_2_ showed a positive gradient increase in the risk of SGA, with quartiles of exposure in the third trimester ranging from 1.587 to 2.375. Referring to the first quartile, the effects of SO_2_ increased with the successive quartiles 2, 3, and 4 during the first and third trimesters.

**Table 2:** Crude and adjusted ORs (95% CIs) for SGA associated with every 1 μg/m^3^ increase in air pollutants

Exposure	Crude			Adjusted		
	OR^a^	95% CI	*P*	*a*OR^b^	95% CI	*P*
PM_10_						
Entire pregnancy	1.692	1.384–2.042	< 0.001	1.055	1.035–1.076	< 0.001
Trimester 1	0.817	0.753–0.886	< 0.001	0.979	0.971–0.987	< 0.001
Trimester 2	1.138	1.062–1.219	< 0.001	1.013	1.006–1.020	< 0.001
Trimester 3	1.184	1.116–1.255	< 0.001	1.018	1.012–1.024	< 0.001
PM_2.5_						
Entire pregnancy	2.179	1.644–2.917	< 0.001	1.084	1.053–1.116	< 0.001
Trimester 1	0.792	0.722–0.877	< 0.001	0.976	0.966–0.986	< 0.001
Trimester 2	1.207	1.094–1.318	< 0.001	1.019	1.009–1.028	< 0.001
Trimester 3	1.219	1.127–1.331	< 0.001	1.022	1.013–1.030	< 0.001
NO_2_						
Entire pregnancy	1.020	0.638–1.629	0.947	1.000	0.953–1.049	0.913
Trimester 1	0.665	0.580–0.776	< 0.001	0.959	0.945–0.973	< 0.001
Trimester 2	1.184	0.990–1.411	0.065	1.016	0.998–1.035	0.079
Trimester 3	1.424	1.195–1.692	< 0.001	1.037	1.019–1.056	< 0.001
SO_2_						
Entire pregnancy	1.629	0.722–3.707	0.238	1.051	0.968–1.141	0.253
Trimester 1	0.319	0.224–0.449	< 0.001	0.887	0.857–0.919	< 0.001
Trimester 2	1.357	0.951–1.913	0.091	1.029	0.993–1.066	0.120
Trimester 3	2.814	2.061–3.873	< 0.001	1.117	1.082–1.153	< 0.001

^a^Crude ORs of air pollutants

^b^Adjusted ORs for covariates, including maternal age, parity, gestational hypertension, gestational diabetes mellitus, delivery mode, and infant sex

**Table 3:** Estimated ORs with 95% CIs of the risk of SGA for each IQR of air pollutants during each pregnancy period compared with the reference group (lowest quartile)

Pollutant	IQR^a^	Entire pregnancy		Trimester 1		Trimester 2		Trimester 3	
		OR^b^ (95% CI)	*P*	OR (95% CI)	*P*	OR (95% CI)	*P*	OR (95% CI)	*P*
PM_10_	Q1	–		–		–		–	
	Q2	3.210 (2.124–4.852)	< 0.001	0.858 (0.639–1.152)	0.308	1.012 (0.710–1.443)	0.946	1.375 (0.736–2.019)	0.104
	Q3	3.241 (2.144–4.899)	< 0.001	0.521 (0.373–0.727)	< 0.001	1.224 (0.873–1.716)	0.242	1.942 (1.353–2.786)	< 0.001
	Q4	3.038 (2.004–4.604)	< 0.001	0.475 (0.338–0.667)	< 0.001	1.590 (1.148–2.202)	0.005	2.461 (1.732–3.498)	< 0.001
PM_2.5_	Q1	–		–		–		–	
	Q2	3.167 (2.094–4.789)	< 0.001	0.853 (0.635–1.147)	0.293	1.240 (0.868–1.773)	0.237	1.563 (1.078–2.266)	0.018
	Q3	3.159 (2.088–4.780)	< 0.001	0.570 (0.410–0.793)	0.001	1.398 (0.987–1.980)	0.059	1.665 (1.154–2.401)	0.006
	Q4	3.151 (2.083–4.768)	< 0.001	0.475 (0.337_0.670)	< 0.001	1.772 (1.267–2.478)	0.001	2.385 (1.681–3.384)	< 0.001
NO_2_	Q1	–		–		–		–	
	Q2	0.678 (0.483–0.951)	0.024	0.789 (0.587–1.061)	0.116	0.666 (0.464–0.955)	0.027	1.993 (1.369–2.903)	< 0.001
	Q3	0.786 (0.569–1.085)	0.143	0.452 (0.321–0.637)	< 0.001	0.855 (0.612–1.194)	0.358	2.052 (1.412–2.981)	< 0.001
	Q4	0.936 (0.686–1.277)	0.678	0.504 (0.363–0.700)	< 0.001	1.454 (1.072–1.972)	0.016	2.375 (1.646–3.426)	< 0.001
SO_2_	Q1	–		–		–		–	
	Q2	0.997 (0.711–1.397)	0.984	0.889 (0.669–1.181)	0.418	1.277 (0.917–1.779)	0.148	1.587 (1.046–2.410)	0.030
	Q3	1.046 (0.752–1.455)	0.790	0.417 (0.297–0.586)	< 0.001	1.022 (0.723–1.445)	0.902	3.138 (2.140–4.063)	< 0.001
	Q4	1.135 (0.822–1.568)	0.442	0.339 (0.235–0.490)	< 0.001	1.309 (0.942–1.819)	0.109	3.074 (2.093–4.515)	< 0.001

^a^Q1: The first quartile; Q2: The second quartile; Q3: The third quartile; Q4: The fourth quartile

^b^Adjusted for maternal age, parity, gestational hypertension, gestational diabetes mellitus, delivery mode, and infant sex

**Fig. 3:**
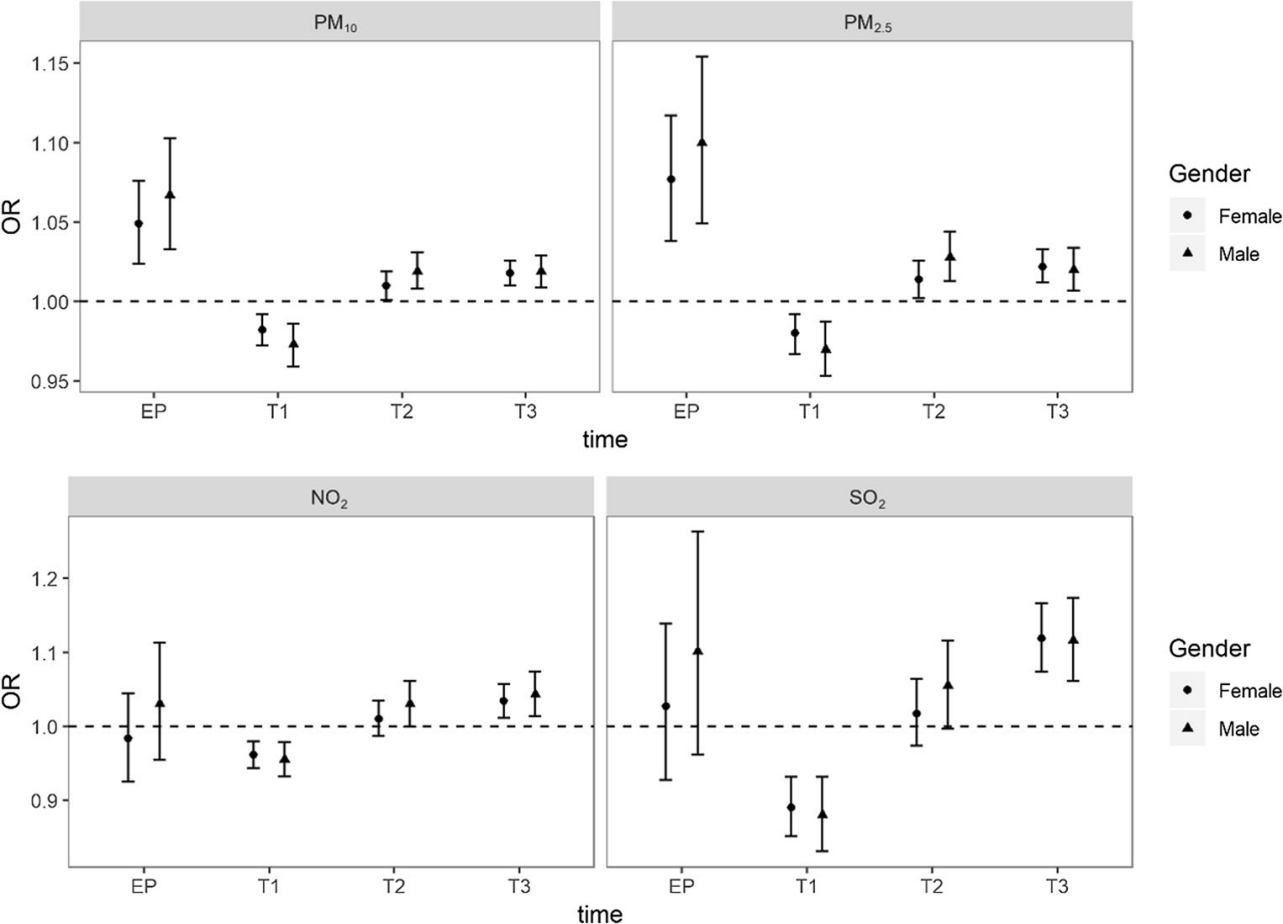
The estimated ORs of each air pollutant with 95% CIs during different pregnancy periods stratified by sex adjusted for maternal age, parity, gestational hypertension, gestational diabetes mellitus, delivery mode, and infant sex. EP: entire pregnancy, T1: the first trimester, T2: the second trimester, and T3: the third trimester

In the stratified analyses by sex, the effects of air pollution exposure were stronger on male infants (Fig. 3). Exposure to PM_10_ and PM_2.5_ was statistically significant during each pregnancy period as pooled infants. The ORs with 95% CIs of PM_10_ were 1.067 (95% CI 1.033–1.103) for males and 1.049 (95% CI 1.024–1.076) for females during the entire pregnancy. The ORs with 95% CIs of PM_2.5_ were 1.100 (95% CI 1.049–1.154) for males and 1.077 (95% CI 1.038–1.117) for females during the entire pregnancy. The effects of NO_2_ and SO_2_ for the two sexes were still statistically significant during the first and third trimesters. The variation between male and female infants during the third trimester was small, and the ORs with 95% CIs of male and female infants were 1.019 (95% CI 1.009–1.029) and 1.018 (95% CI 1.010–1.026) for PM_10_, 1.020 (95% CI 1.007–1.034) and 1.022 (95% CI 1.012–1.033) for PM_2.5_, 1.043 (95% CI 1.013–1.074) and 1.034 (95% CI 1.011–1.057) for NO_2_, and 1.116 (95% CI 1.061–1.174) and 1.119 (95% CI 1.009–1.029) for SO_2_.

**Fig. 4:**
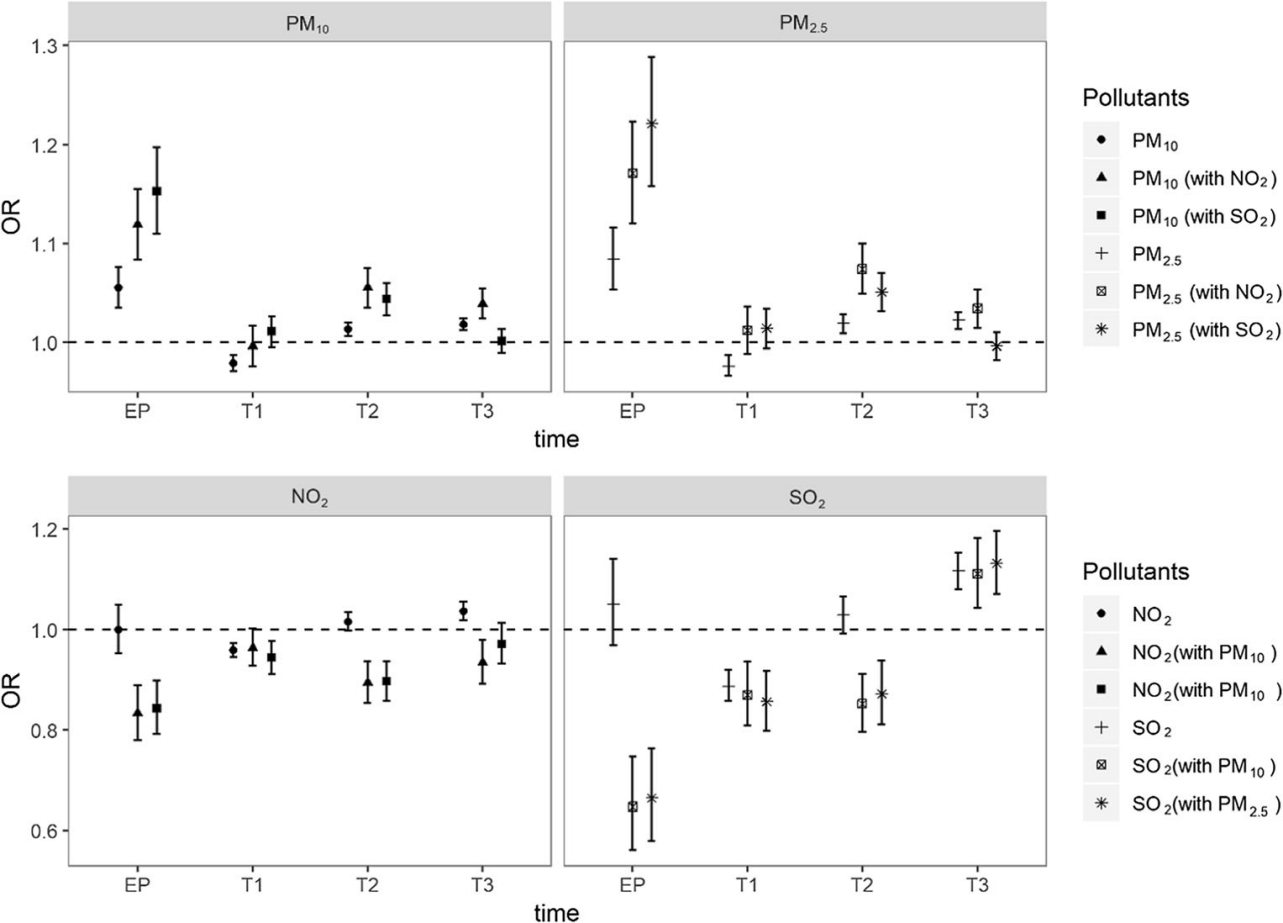
The estimated ORs for the risk of SGA with 95% CIs in single-pollutant models and multi-pollutant models adjusted for maternal age, parity, gestational hypertension, gestational diabetes mellitus, delivery mode, and infant sex. EP: entire pregnancy, T1: the first trimester, T2: the second trimester, T3: the third trimester

Two pollutant models were developed to examine the potential confounding effects of other pollutants in our model (Fig. 4). Compared with a single-pollutant model for PM_10_ and PM_2.5_ (1.055 1.035–1.076 for PM_10_ and 1.084 1.053–1.116 for PM_2.5_), the effects of particulate matter were strengthened when adjusted for NO_2_ and SO_2_. The ORs with 95% CIs of PM_10_ were 1.119 (95% CI 1.084–1.155) adjusted for NO_2_ and 1.153 (95% CI 1.110–1.197) adjusted for SO_2_ during the entire pregnancy. Similar to PM_10_, the ORs with 95% CI of PM_2.5_ were 1.171 (95% CI 1.120–1.223) adjusted for NO_2_ and 1.221 (95% CI 1.158–1.288) adjusted for SO_2_. However, the effects of NO_2_ and SO_2_ were not stable, as some of their effects were strengthened, while some were attenuated due to the collinearity between pollutants. The seasonal analysis showed statistically significant associations between air pollution and SGA in summer and winter (Table S3).

## Discussion

This was a cross-sectional study conducted in Huangshi in the eastern Hubei province. Delivery data was collected from the Huangshi Maternity and Children’s Health Hospital for the entire year of 2017. In this study, the incidence of SGA was 6.7%, which was lower than the percentile used to define this category (Wang et al. 2019). In this research, exposure to PM_10_ and PM_2.5_ was adversely associated with the risk of SGA during all pregnancy periods. The effects of NO_2_ and SO_2_ exposure were less remarkable, but still significant during the first and third trimesters. This is the first population-based cross-sectional study that has investigated the association between SGA and air pollution in central China. The population selected in this study accounted for more than one-third of the infants delivered in Huangshi in the entire year. Therefore, the population was comparatively representative. Compared with other big cities, Huangshi is a prefecture-level city with a lower residential moving rate, indicating that all of the participants had consistent maternal exposure to pollutants.

With the exception of the first trimester, an increased risk of SGA associated with higher levels of PM_10_ and PM_2.5_ exposure was observed during the other two trimesters. Despite that many previous studies have examined the effects of PM_10_ on SGA, the results have remained inconclusive. While studies have provided multiple evidences for an increased risk of SGA with exposure to PM_10_ in specific trimesters (Ha et al. 2017; Hansen et al. 2007; Le et al. 2012; Mannes et al. 2005), some studies have found a null association between SGA and PM_10_ (Brauer et al. 2008; Capobussi et al. 2016; Kimberly et al. 2014; Madsen et al. 2010; Wang et al. 2019). Unlike PM_10_, many previous studies have indicated the adverse effects of PM_2.5_ on SGA, as found in this study (Mannes et al. 2005; Percy et al. 2019; Ritz et al. 2007; Stieb et al. 2016), but these studies did not reach a consensus on trimester-specific effects. Identified exposure windows for PM_2.5_ exposure and SGA have been reported in the first trimester (Rich et al. 2009b), the second trimester (Mannes et al. 2005; Wang et al. 2019), the third trimester (Percy et al. 2019; Rich et al. 2009b), and the entire pregnancy period (Stieb et al. 2016).

In addition to the significant relationships during the first trimester, a positive association was found between NO_2_, SO_2_, and SGA during the third trimester. Some studies have found a positive association between NO_2_ exposure and SGA in specific trimesters (Ballester et al. 2010; Stieb et al. 2016; Wang et al. 2019); however, some studies have also reported no evidence of adverse impacts from NO_2_ on SGA (Mannes et al. 2005; Rich et al. 2009a; Ritz et al. 2007; Taylor et al. 2016). Previous studies regarding the association between SO_2_ and SGA have remained inconclusive, with several studies having reported no association, while others have found a weak association (Ha et al. 2017; Le et al. 2012; Rich et al. 2009a). In summary, previous studies have not observed a stable and positive association between NO_2_, SO_2_, and SGA.

The discrepancies among this study and previous studies may be attributed to many factors. First, as mentioned, most studies regarding the association between air pollution and SGA have been conducted in developed areas, and the lifestyles of the study populations, such as nutrition, cooking methods, and medical care, which could vary from those of women in Huangshi. Second, many studies adopted more accurate methods based on residential address to estimate the individual exposure, while this study used the mean concentrations collected from monitory station, suggesting a variability of exposure assessment (Percy et al. 2019; Wang et al. 2019). Third, spatial heterogeneity could result in distinct composition of air pollutants, which means that even pollutants with the same concentrations could result in very different exposures (Bell et al. 2010).

In this study, it was found that exposure to ambient air pollution decreased the risk of SGA during the first trimester, which seems not biologically plausible and inconsistent with previous studies. Most studies have found adverse effects from ambient air pollutants. One potential explanation for this discrepancy is that embryos that were easily injured were aborted or resulted in stillbirths in the early maternal stage of air pollutant exposure. However, in this study, only singleton live births were considered; thus, the population that was selected in this study was partially biased (Percy et al. 2019).

During the third trimester, the risk of SGA grew greater than during other periods as the concentration of air pollutants increased. The third trimester is a very important period for fetal growth because the most rapid fetal development occurs in this period (Grantz et al. 2018). Hence, the fetus may be more vulnerable than during other periods. The biological mechanism of the adverse impacts from air pollution on newborns remains unclear. It is assumed that air pollutants could alter fetal growth by causing oxidative stress or inflammation, reducing placental exchange of nutrients and gases, negatively affecting placental growth, fostering endocrine disruption, or causing negative maternal health effects (Kannan et al. 2006). Another hypothesis is that air pollution can affect the mitochondria. For instance, exposure to NO_2_ causes damage to mitochondrial DNA, which is related to infant birth weight (Clemente et al. 2016). Prenatal PM_10_ exposure has been associated with placental mitochondrial alterations, which may both reflect and intensify oxidative stress production (Janssen et al. 2012).

In this time-stratified analysis, it was found that exposure to air pollution in summer and winter was associated with SGA morbidity. The increased risk of SGA in summer may be the result of high-temperature exposure. Heat stress could cause damage to the antioxidant defense system and result in a larger secretion amount of oxytocin (Forgati et al. 2017), which could have a negative impact on maternal health and fetal growth. The association in winter could be explained by a deficiency in vitamin D. Vitamin D has been found to be negatively correlated to SGA risk (Wang et al. 2018), and the level of vitamin D in winter in China is much lower than that in summer (Yang and Zhang 2013).

In the subgroup analysis, it was found that the effects of environmental exposure to air pollution could be modified by infant sex, which has been mentioned in many previous studies (Lee et al. 2014; Liu et al. 2018; Taylor et al. 2016; Wainstock et al. 2014). Lee et al. (2014) suggested that prenatal exposure to bisphenol A played a different role in birth weights between male infants and female infants in a multi-center birth cohort study from Korea (Lee et al. 2014). A study in Shanghai, China, found that maternal exposure to household air pollution could contribute to adverse birth outcomes in boys but not in girls (Liu et al. 2018). However, a population-based study in the UK showed that moderate prenatal cadmium exposure was associated with lower birth weights in girls but not in boys (Taylor et al. 2016). In addition, one retrospective cohort study reported that exposure to prenatal maternal stress was a risk factor only in female fetuses (Wainstock et al. 2014). These findings suggest that sexual differences may alter the effects of the external environment, and variations between sexes should be noticed. Some studies have attempted to provide biologically plausible explanations for these variations. Male fetuses were assumed to have greater potential susceptibility to a pro-inflammatory environment during pregnancy. In vitro studies have shown that when infected, cells from pregnancies with a male fetus were observed to have an increase in production of pro-inflammatory cytokines, including tumor necrosis factor and prostaglandin synthase, and had a smaller quantity of anti-inflammatory cytokines and prostaglandin dehydrogenase compared with female infants (Al-Qaraghouli and Fang 2017; Challis et al. 2013; Liu et al. 2018).

The quartile assessment showed that the risk of SGA under exposure to PM_10_ and PM_2.5_ was not distinct during the entire pregnancy. However, the trimester-specific analysis showed that the effects of PM_10_ and PM_2.5_ became stronger with increasing concentrations, suggesting that the risk for SGA could increase without a threshold effect of pollutants as the concentration of PM_10_ and PM_2.5_ increased. The associations between NO_2_, SO_2_, and SGA were not as stable as particulate matter, but an OR increase in SGA still was observed per IQR increase during the third trimester. Previous studies have not reached a consistent conclusion on how air pollutants with different quartiles affect SGA, as they have reported no significantly positive associations or even a decreased risk of SGA for higher levels of PM_10_ and NO_2_ (Eh et al. 2012; Hansen et al. 2007; Wu et al. 2018). In summary, whether higher concentrations of air pollutants will lead to increased risk of SGA requires further research.

Several limitations should be considered when interpreting this research. The accurate address of each pregnant woman was not acquired. Therefore, the mean concentration of each air quality monitoring station in Huangshi was used to replace individual exposure. The lack of an accurate maternal exposure model may lead to some misclassifications due to the fact that air pollutant concentration levels are not homogenous across different locations. In this cross-sectional study, only pregnancy data for the year of 2017 was collected, which is a research period restriction. In further studies, a larger cohort that covers several continuous years needs to be collected to observe the effect of air pollution on SGA based on a retrospective study. Due to the hospital location in central Huangshi, the population recruited was primarily from the urban city; thus, the condition of rural areas could not be presented in this research.

In this study, although SGA was found to have a 2% to 10% increased risk when exposed to air pollution during the third trimester, considering the high concentration of air pollutants in Huangshi, exposure to ambient air pollution is still a severe risk factor for fetal growth restriction. Therefore, these findings are significant enough to encourage the government to take actions to reduce air pollutant emissions to improve the health condition of pregnant women and their infants. It is worthwhile to continue air quality improvements for the prevention of SGA infants and to reduce the costs associated with SGA infants later in life.

## Conclusion

In this cross-sectional study, it was found that maternal exposure to air pollution was significantly associated with SGA. Overall, the adverse effects of PM_10_ and PM_2.5_ were stronger than gaseous pollutants, including NO_2_ and SO_2_. In addition, the third trimester was found to be the most vulnerable period for fetuses when exposed to air pollution. These findings can provide a basis for air quality management policies and the promotion of neonatal health. More studies in other prefecture cities with high levels of air pollutants should be conducted, which conform to this study in the future.

## Electronic supplementary material

ESM 1 (PDF 117 kb)

## References

[CR1] Al-Qaraghouli M, Fang YMV (2017). Effect of fetal sex on maternal and obstetric outcomes. Frontiers in Pediatrics.

[CR2] Ballester F, Estarlich M, Iñiguez C, Llop S, Ramón R, Esplugues A, Lacasaña M, Rebagliato M (2010). Air pollution exposure during pregnancy and reduced birth size: a prospective birth cohort study in Valencia, Spain. Environmental Health: a Global Access Science Source.

[CR3] Basso O, Wilcox AJ, Weinberg (2006). Birth weight and mortality: causality or confounding?. American Journal of Epidemiology.

[CR4] Bell ML, Kathleen B, Keita E, Gent JF, Hyung Joo L, Petros K, Leaderer BP (2010). Prenatal exposure to fine particulate matter and birth weight: variations by particulate constituents and sources. J Epidemiology.

[CR5] Brauer M, Lencar C, Tamburic L, Koehoorn M, Demers P, Karr C (2008). A cohort study of traffic-related air pollution impacts on birth outcomes. Environmental health perspectives.

[CR6] Browne VA, Julian CG, Lillian TJ, Darleen CR, Enrique V, Moore LG (2015). Uterine artery blood flow, fetal hypoxia and fetal growth. J Philosophical Transactions of the Royal Society B Biological Sciences.

[CR7] Capobussi M, Tettamanti R, Marcolin L, Piovesan L, Bronzin S, Gattoni ME, Polloni I, Sabatino G, Tersalvi CA, Auxilia F, Castaldi S (2016). Air pollution impact on pregnancy outcomes in Como, Italy. Journal of Occupational and Environmental.

[CR8] Catalano R, Karasek D, Gemmill A, Falconi A, Goodman J, Magganas A, Hartig T (2014). Very low birthweight: dysregulated gestation versus evolutionary adaptation. Social Science & Medicine.

[CR9] Challis J, Newnham J, Petraglia F, Yeganegi M, Bocking A (2013). Fetal sex and preterm birth. J Placenta.

[CR10] Clayton PE, Cianfarani S, Czernichow P, Johannsson G, Rapaport R, Rogol A (2007). Management of the child born small for gestational age through to adulthood: a consensus statement of the International Societies of Pediatric Endocrinology and the Growth Hormone Research Society. The Journal of Clinical Endocrinology & Metabolism.

[CR11] Clemente DBP, Casas M, Vilahur N, Begiristain H, Bustamante M, Carsin AE, Fernández MF, Fierens F, Gyselaers W, Iñiguez C, Janssen BG, Lefebvre W, Llop S, Olea N, Pedersen M, Pieters N, Santa Marina L, Souto A, Tardón A, Vanpoucke C, Vrijheid M, Sunyer J, Nawrot TS (2016). Prenatal ambient air pollution, placental mitochondrial DNA content, and birth weight in the INMA (Spain) and ENVIRONAGE (Belgium) birth cohorts. Environmental Health Perspectives.

[CR12] Ding G, Tian Y, Zhang Y, Pang Y, Zhang J, Zhang J (2013). Application of a global reference for fetal-weight and birthweight percentiles in predicting infant mortality. BJOG.

[CR13] Eh VDH, Pierik FH, De KY, Willemsen SP, Hofman A, van Ratingen SW, Zandveld PY, Mackenbach JP, Steegers EA, Miedema HMJEHP (2012). Air pollution exposure during pregnancy, ultrasound measures of fetal growth, and adverse birth outcomes: a prospective cohort study. Environ Health Perspect.

[CR14] Forgati M, Kandalski PK, Herrerias T, Zaleski T, Machado C, Souza MRDP, Donatti L, Forgati M, Kandalski PK, Herrerias T (2017). Effects of heat stress on the renal and branchial carbohydrate metabolism and antioxidant system of Antarctic fish. J Comp Physiol B.

[CR15] Grantz KL, Hediger ML, Liu D, Buck Louis GM (2018). Fetal growth standards: the NICHD fetal growth study approach in context with INTERGROWTH-21st and the World Health Organization Multicentre Growth Reference Study. Am J Obstet Gynecol.

[CR16] Ha S, Zhu Y, Liu D, Sherman S, Mendola P (2017). Ambient temperature and air quality in relation to small for gestational age and term low birthweight. Environmental research.

[CR17] Hansen C, Neller A, Williams G, Simpson R (2007). Low levels of ambient air pollution during pregnancy and fetal growth among term neonates in Brisbane, Australia. Environmental Research.

[CR18] Janssen BG, Munters E, Pieters N, Smeets K, Cox B, Cuypers A, Fierens F, Penders J, Vangronsveld J, Gyselaers W, Nawrot TS (2012). Placental mitochondrial DNA content and particulate air pollution during in utero life. Environmental Health Perspectives.

[CR19] Kannan S, Misra DP, Dvonch JT, Krishnakumar A (2006). Exposures to airborne particulate matter and adverse perinatal outcomes: a biologically plausible mechanistic framework for exploring potential effect modification by nutrition. Environmental Health Perspectives.

[CR20] Khambalia AZ, Algert CS, Bowen JR, Collie RJ, Roberts CL (2017). Long-term outcomes for large for gestational age infants born at term. J Paediatr Child Health.

[CR21] Kildea SV, Gao Y, Rolfe M, Boyle J, Tracy S, Barclay LM (2017). Risk factors for preterm, low birthweight and small for gestational age births among Aboriginal women from remote communities in Northern Australia. Women and Birth.

[CR22] Kimberly H, Roseanne MN, Philip B, Colin S, Raymond AJ (2014). Air pollution exposure and adverse pregnancy outcomes in a large UK birth cohort: use of a novel spatio-temporal modelling technique. Scand J Work Environ Health.

[CR23] Lamichhane DK, Leem JH, Lee JY, Kim HC (2015). A meta-analysis of exposure to particulate matter and adverse birth outcomes. Environmental Health & Toxicology.

[CR24] Le HQ, Batterman SA, Wirth JJ, Wahl RL, Hoggatt KJ, Sadeghnejad A, Hultin ML, Depa M (2012). Air pollutant exposure and preterm and term small-for-gestational-age births in Detroit, Michigan: long-term trends and associations. Environment International.

[CR25] Lee B-E, Park H, Hong Y-C, Ha M, Kim Y, Chang N, Kim B-N, Kim YJ, Yu S-D, Ha E-H (2014). Prenatal bisphenol A and birth outcomes: MOCEH (Mothers and Children’s Environmental Health) study. International Journal of Hygiene and Environmental Health.

[CR26] Lefebvre G, Samoilenko M (2017). Correction to: On the use of the outcome variable “small for gestational age” when gestational age is a potential mediator: a maternal asthma perspective. BMC Medical Research Methodology.

[CR27] Li X, Huang S, Jiao A, Yang X, Yun J, Wang Y, Xue X, Chu Y, Liu F, Liu Y (2017). Association between ambient fine particulate matter and preterm birth or term low birth weight: an updated systematic review and meta-analysis ☆. Environmental Pollution.

[CR28] Liu W, Huang C, Cai J, Wang X, Zou Z, Sun C (2018). Household environmental exposures during gestation and birth outcomes: a cross-sectional study in Shanghai, China. Science of The Total Environment.

[CR29] Madsen C, Gehring U, Erik Walker S, Brunekreef B, Stigum H, Næss Ø, Nafstad P (2010). Ambient air pollution exposure, residential mobility and term birth weight in Oslo, Norway. Environmental Research.

[CR30] Mannes T, Jalaludin B, Morgan G, Lincoln D, Sheppeard V, Corbett S (2005). Impact of ambient air pollution on birth weight in Sydney, Australia. Occupational and Environmental Medicine.

[CR31] Michikawa T, Morokuma S, Fukushima K, Kato K, Nitta H, Yamazaki S (2017). Maternal exposure to air pollutants during the first trimester and foetal growth in Japanese term infants. Environmental Pollution.

[CR32] Percy Z, DeFranco E, Xu F, Hall ES, Haynes EN, Jones D, Muglia LJ, Chen A (2019). Trimester specific PM2.5 exposure and fetal growth in Ohio, 2007–2010. Environmental Research.

[CR33] Qian Z, Liang S, Yang S, Trevathan E, Huang Z, Yang R, Wang J, Hu K, Zhang Y, Vaughn M, Shen L, Liu W, Li P, Ward P, Yang L, Zhang W, Chen W, Dong G, Zheng T, Xu S, Zhang B (2016). Ambient air pollution and preterm birth: a prospective birth cohort study in Wuhan, China. Int J Hyg Environ Health.

[CR34] Rich DQ, Demissie K, S-E L, Kamat L, Wartenberg D, Rhoads GG (2009). Ambient air pollutant concentrations during pregnancy and the risk of fetal growth restriction. J Journal of Epidemiology, Health C.

[CR35] Rich DQ, Demissie K, Lu SE, Kamat L, Wartenberg D, Rhoads GG (2009). Ambient air pollutant concentrations during pregnancy and the risk of fetal growth restriction. Journal of epidemiology and community health.

[CR36] Ritz B, Wilhelm M, Hoggatt KJ, Ghosh JKC (2007). Ambient air pollution and preterm birth in the environment and pregnancy outcomes study at the University of California, Los Angeles. American Journal of Epidemiology.

[CR37] Schlesinger RB, Kunzli N, Hidy GM, Gotschi T, Jerrett M (2006). The health relevance of ambient particulate matter characteristics: coherence of toxicological and epidemiological inferences. Inhalation Toxicology.

[CR38] Stieb DM, Chen L, Hystad P, Beckerman BS, Jerrett M, Tjepkema M, Crouse DL, Omariba DW, Peters PA, van Donkelaar A, Martin RV, Burnett RT, Liu S, Smith-Doiron M, Dugandzic RM (2016). A national study of the association between traffic-related air pollution and adverse pregnancy outcomes in Canada, 1999–2008. Environmental Research.

[CR39] Taylor CM, Golding J, Emond AM (2016). Moderate prenatal cadmium exposure and adverse birth outcomes: a role for sex-specific differences?. Paediatric and perinatal epidemiology.

[CR40] Wainstock T, Shoham-Vardi I, Glasser S, Anteby E, Lerner-Gev LJ (2014). Fetal sex modifies effects of prenatal stress exposure and adverse birth outcomes. Stress.

[CR41] Wang H, Xiao Y, Zhang L, Gao Q (2018). Maternal early pregnancy vitamin D status in relation to low birth weight and small-for-gestational-age offspring. The Journal of Steroid Biochemistry and Molecular Biology.

[CR42] Wang Q, Benmarhnia T, Li C, Knibbs LD, Bao J, Ren M, Zhang H, Wang S, Zhang Y, Zhao Q, Huang C (2019). Seasonal analyses of the association between prenatal ambient air pollution exposure and birth weight for gestational age in Guangzhou, China. Science of The Total Environment.

[CR43] WHO (2005) Air quality guidelines - global update 2005. Accessed 25 April 2019

[CR44] Wu J, Ren C, Delfino RJ, Chung J, Wilhelm M, Ritz B (2009). Association between local traffic-generated air pollution and preeclampsia and preterm delivery in the south coast air basin of California. Environmental health perspectives.

[CR45] Wu H, Jiang B, Geng X, Zhu P, Liu Z, Cui L, Yang L (2018). Exposure to fine particulate matter during pregnancy and risk of term low birth weight in Jinan, China, 2014–2016. International Journal of Hygiene and Environmental Health.

[CR46] Yang L, Zhang W (2013). Seasonal vitamin D and bone metabolism in women of reproductive age in urban Beijing. Asia Pacific Journal of Clinical Nutrition.

[CR47] Zhan Changlin, Zhang Jiaquan, Zheng Jingru, Yao Ruizhen, Wang Ping, Liu Hongxia, Xiao Wensheng, Liu Xianli, Cao Junji (2017). Characterization of carbonaceous fractions in PM2.5 and PM10 over a typical industrial city in central China. Environmental Science and Pollution Research.

[CR48] Zhu L, Zhang R, Zhang S, Shi W, Chen C (2015). Chinese neonatal birth weight curve for different gestational age. Zhonghua er ke za zhi. Chinese Journal of Pediatrics.

