# Supplementary material for: Association between ambient air pollution exposure and infants small for gestational age in Huangshi, China: a cross-sectional study
Source: Environ Sci Pollut Res Int. 2019 Sep 6;26(31):32029–39. doi: 10.1007/s11356-019-06268-7 (PMC6875112; doi:10.1007/s11356-019-06268-7)
Supplement: Supplementary file 1 — (PDF 117 kb) [file 11356_2019_6268_MOESM1_ESM.pdf]

The association between ambient air pollution exposure and infant small for gestational age in Huangshi, China: a cross–sectional study.

Journal: Environmental Science and Pollution Research

Jiayuan Hao[1], Faxue Zhang[1], Dieyi Chen[1], Yanyun Liu[2], Lina Liao[2], Cui Shen[2], Tianyu Liu[1], Jingling Liao[3], Lu Ma[1,4*]

1 Department of Healthcare Management, School of Health Sciences, Wuhan University, Wuhan 430071, China;

2 Huangshi Maternity and Children's Health Hospital of Edong Healthcare Group, Hubei Province, China;

3 Department of Public Health, Wuhan University of Science and Technology School of Medicine, Wuhan;

4 Global Health Institute, Wuhan University, Wuhan 430071, China

*Correspondence: Lu Ma(malu@whu.edu.cn); Department of Healthcare Management, School of Health Sciences, Wuhan University, Wuhan 430071, China; Tel.: +86-27-6875-9901

Supplementary materials:

Table S1. The correlation of air pollutants during entire pregnancy

	PM_{10}	$PM_{2.5}$	NO_2	SO_2
PM_{10}	1.000			
$PM_{2.5}$	0.998	1.000		
NO_2	0.660	0.644	1.000	
SO_2	0.804	0.788	0.835	1.000

Table S2. Characteristics of pollutant concentration (μg/m^3)

Pollutants	Mean	SD	Max	75th	50th	25th	Min	IQR
PM_{10}	86.8	41.0	255	116	82	37	24	79
$PM_{2.5}$	55.3	28.3	181	75	51	21	12	54
NO_2	36.5	14.1	82	45	30	17	10	28
SO_2	17.6	8.6	54	25	16	6	3	19
